# Post-acute CoVid-19 syndrome (PACS) linked cardiovascular symptoms

**DOI:** 10.6026/973206300200412

**Published:** 2024-05-31

**Authors:** Allen Khakshooy, Francesco Chiappelli

**Affiliations:** 1 Department of Internal Medicine, Valley Hospital Medical Center, Las Vegas, NV 89106, USA; 2 Dental Group of Sherman Oaks, Sherman Oaks, CA 91403, USA; 3 UCLA Center for the Health Sciences, Los Angeles, CA 90095, USA

**Keywords:** Post-acute CoViD-19 syndrome (PACS), long CoViD-19, cardiovascular disease, heart failure (HF), renin-angiotensin-aldosterone system (RAAS), left ventricular ejection fraction (LVEF), angiotensin II receptor-neprilysin inhibitors (ARNi)

## Abstract

Officials have marked the end of the CoVid-19 pandemic, yet we continue to learn more about the SARS-CoV2 virus itself and its lasting multidimensional effects after acute infection. Long COVID, or the post-acute CoViD-19 syndrome (PACS), manifests as a wide range of prolonged physical, mental, and emotional symptoms over at least 1 to 12 months after SARS-CoV2 infection. Here, we describe certain pervasive clinical consequences of PACS on the cardiovascular system, and insight on the potentially improved prognoses in heart failure patients.

## Background:

Colloquially referred to as Long COVID, the post-acute CoViD-19 syndrome (PACS) consists in a wide range of symptoms that occur at least 1 to 12 months after acute SARS-CoV2 infection [1, 2]. At the cellular level, the principal mediators of the immune system, CD4+/CD8+ T cells and B cells, act in harmony with cytokines to mount an appropriate immune response to viral infection. We have described the inability of prolonged CD8+ T cell activation in the face of high-grade chronic SARS-CoV2 infection, forcing a state of T cell exhaustion and translating to greater disease severity [3]. The constellation of prolonged symptoms in PACS can be any single or combination of physical, mental, and emotional pathologies, the most common symptoms including fatigue, dyspnea, myalgia (including chest pain), insomnia, and anosmia [1-4]. PACS also has serious pervasive clinical consequences on the cardiovascular health.

## Cardiovascular Sequelae of Acute CoViD-19:

The clinical manifestations of an acute CoVid-19 pathology following SARS-CoV2 infection on the cardiovascular system are well documented. The symptomatology ranges from no symptoms to dyspnea, chest pain, and palpitations. The most common acute cardiac manifestations include myocardial infarction, peri-myocarditis, arrhythmias, Takatsubo or stress cardiomyopathy, both left and right heart failure, and cardiogenic shock. Diagnoses are supported by elevated troponin levels, ECGs, 24h-Holter monitor, TTE, angiogram, and even cardiac MRI [2, 4].

An even larger population of patients exhibiting cardiovascular sequelae present without apparent cardiac-related symptoms. Acute myocardial injury secondary to the severe disease burden brought about by the acute viral infection is often the only manifestation of cardiac pathology. In the absence of other signs, symptoms, or laboratory evidence, it can clinically present as an isolated elevation in plasma troponin levels, which are a biomarker of cardiomyocytes death and clinically diagnosed as a type II non-ST segment myocardial infarction (NSTEMI) [2-4].

## Cardiovascular Sequelae of PACS:

The persistence of symptoms or development of new symptoms beyond acute infection begins to heighten suspicion of PACS in the appropriate clinical setting. There is strong to support the increased incidence of cardiovascular diseases in recovering patients 4 weeks prior, and up to 12 months after acute CoVid-19 [4, 5]. The risk of cardiovascular sequelae of PACS was evident even in those without conventional risk factors and independent of traditional demographics [5]. An elevated risk is associated with age, sex, and other comorbidities [6]. Acute CoViD-19-recovering patients are at increased of ischemic heart disease, heart failure, arrhythmias, heart failure, and a wide array of hypercoagulable thrombotic disorders [5, 7].

## Heart Failure and PACS:

In general, a wide variety of acute infections can decompensate preexisting heart failure, while also elucidating new-onset heart failure. Infectious etiologies may also be the primary cause of new-onset, acute heart failure. There are several viruses (e.g., Influenza virus A & B, Adenovirus) that are well known to be the primary cause of heart failure, initially beginning as an acute myocarditis, and progressing to a dilated cardiomyopathy of fast increasing severity. Acute myocarditis from SARS-CoV2 has also been a force to be reckoned with during the pandemic [8]. As a secondary cause, acute viral infections can act as the impetus to an already long history of risk factors that bring about heart failure. Indeed, CoVid-19 and PACS can pose a multitude of both direct and indirect factors leading to the new-onset or worsening of heart failure [9].

## Independent of CoVid-19 or PACS, the treatment of heart failure is centered on two main principles:

1.Symptoms reduction

2.Underlying cardiac dysfunction responsible for said symptoms

The intricacies of ejection fraction - reduced vs preserved-and phase-systole vs diastole - will be saved for a more focused study elsewhere. Nevertheless, common to all forms of heart failure therapies is the reduction of symptoms caused volume overload (more specifically, fluid retention) and improving cardiac function, often measured as cardiac output. The current guideline-directed medical therapies for the treatment of heart failure (HF-GDMT) include a single and or combination of: angiotensin-converting enzyme inhibitors (ACEIs), angiotensin II-receptor blockers (ARBs), neprilysin inhibitors, mineralocorticoid receptor antagonists (MRAs), beta blockers and sodium-glucose co-transporter 2 (SGLT-2) inhibitors.

The sequence, optimization, duration, and overall management of these medications are under the purview of clinicians and cardiologists alike. Our focus will specifically be on the medications that act to antagonize the renin-angiotensin- aldosterone system (RAAS), specifically ACEIs, ARBs, and neprilysin inhibitors. The RAAS is a complex system of enzymes and hormones that act synchronously through multiple organs within the vasculature to control blood volume and systemic vascular resistance. These actions translate to volume status and blood pressure, which are critical components of both the diagnosis and treatment of heart failure.

## Use of RAAS Antagonists in PACS-related HF:

Early in the CoVid-19 pandemic, it was suggested that the use of RAAS antagonists may worsen acute SARS-CoV2 infection. As we have described elsewhere [1, 3], the spike protein of SARS-CoV2 is the principal ligand to ACE2 receptor which, along with other recently identified receptors such as CD209 [10] and RAGE [11], allows for receptor-mediated viral entry into cells. We also learned of the strong, positive correlation between receptor activation and disease severity [10-12]. Thus, as the most abundant of these receptors in our organs, ACE2 was well scrutinized. Specifically, the upstream inhibition by ACEIs and ARBs was thought to indirectly cause up regulation of the ACE2 receptors, thereby leading to a more profound systemic immune response. However, these patients were found to have no statistically significant difference in all-cause mortality in the face of CoViD-19, and the continued use of these inhibitors was encouraged [13].

Another indirect antagonist of the RAAS is neprilysin. Neprilysin is a group of enzymes that physiologically act to degrade natriuretic peptides released by the heart, such as atrial and brain natriuretic peptides (ANP and BNP, respectively). By inhibiting their degradation, the high levels of these peptides cause a physiologic natriuretic and subsequent release of excessive sodium and water. These actions serve to further improve heart failure symptoms of volume overload and reducing adverse remodeling of cardiac tissue. Currently, sacubitril is the only available neprilysin inhibitor available as a part of combination therapy with an ARB, collectively known as angiotensin receptor-neprilysin inhibitors (ARNi). The ARNi combination yields improved prognosis of heart failure patients suffering from CoVid-19 [14], and presumably PACS, with the action of natriuretic peptides implicated as key players [15].

## Protection from RAAS Antagonism:

As described above, there is a growing prevalence of alterations to cardiac tissue in recovering patients that requires continued monitoring [16]. Heart monitoring with transthoracic echocardiography has shown affected patients with evidence of both reduced systolic and diastolic functioning [17]. The consensus regarding the increased incidence of heart failure as a long-term consequence of CoVid-19 is clear [5], and the role our advanced therapies play in the setting of these patients and their associated prognoses is still growing.

## Conclusion:

There exists evidence to suggest significant improvement in heart failure symptoms and cardiac abnormalities, such low left ventricular ejection fraction, in recovering patients with the initiation of traditional HF-GDMT [14, 17]. A four month follow up of post-CoViD conditions found that less than 10% of patients were found to have a left ventricular ejection fraction (LVEF) of <50%, of which none had an LVEF of less than 40% [16]. Of note, many investigations into CoViD-related myocardial injury and cardiac alterations show minimal significance in the larger clinical picture [17, 18, 19]. Taken together, the early initiation and safe advancement of HF-GDMT seems to show better outcomes, decreased mortality, and improved prognosis of heart failure patients suffering from PACS.
